# Supplementary material for: Identification of photoperiod-induced specific miRNAs in the adrenal glands of Sunite sheep (Ovis aries)
Source: Front Vet Sci. 2022 Jul 22;9:888207. doi: 10.3389/fvets.2022.888207 (PMC9354845; doi:10.3389/fvets.2022.888207)
Supplement: Supplementary file 1 [file Data_Sheet_1.ZIP › Supplementary file/miRDeep2 file.zip!/miRDeep2 file/Know_Structure/SPLP42A1/oar-mir-409.pdf]

miRBase precursor : oar-mir-409
Total read count : 14866
oar-miR-409-5p read count : 2976
oar-miR-409-3p read count : 11878
remaining reads : 12

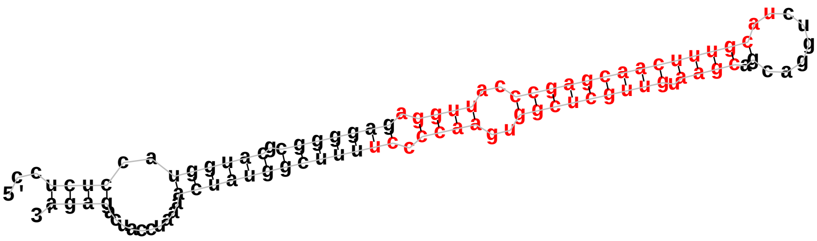

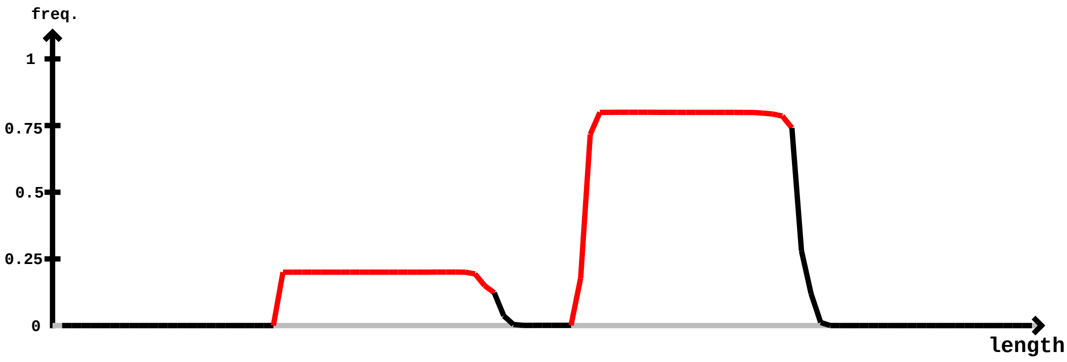

oar-miR-409-5p

oar-miR-409-3p

```
5'- ccucuccaugguacgcggggagagguuacccgagcaacuuugcaucuggacgacgaauguugcucggugaaccccuuuucgguaucaaaauccaucagaga  -3'    exp
    .((((...((((((.(((((((.((((..((((((((((((((........).)))).)))))))))..)))).)))))))))))))).........)))).   reads   mm   sample
    ......................agguuacccgagcaacu..............................................................   1       0    seq
    ......................agguuacccgagcaacuuu............................................................   8       0    seq
    ......................agguuacccgagcaacuuug...........................................................   95      0    seq
    ......................agguuacccgagcaacuuugc..........................................................   653     0    seq
    ......................agguuacccgagcaacuuugca.........................................................   395     0    seq
    ......................agguuacccgagcaacuuugcau........................................................   1291    0    seq
    ......................agguuacccgagcaacuuugcauc.......................................................   482     0    seq
    ......................agguuacccgagcaacuuugcaucu......................................................   49      0    seq
    .......................gguuacccgagcaacuuugcau........................................................   1       0    seq
    .......................gguuacccgagcaacuuugcaucu......................................................   1       0    seq
    ......................................uuugcaucuggacgacgaauguu........................................   4       0    seq
    ......................................uuugcaucuggacgacgaauguug.......................................   1       0    seq
    ......................................uuugcaucuggacgacgaauguugc......................................   1       0    seq
    ........................................ugcaucuggacgacgaauguugcu.....................................   1       0    seq
    ............................................ucuggacgacgaauguugcucg...................................   1       0    seq
    ..............................................uggacgacgaauguugcucggugaaccc...........................   2       0    seq
    .................................................acgacgaauguugcucgguga...............................   1       0    seq
    .................................................acgacgaauguugcucggugaacc............................   1       0    seq
    ....................................................acgaauguugcucggugaac.............................   2       0    seq
    ....................................................acgaauguugcucggugaacc............................   1       0    seq
    .....................................................cgaauguugcucgguga...............................   1       0    seq
    .....................................................cgaauguugcucggugaa..............................   2       0    seq
    .....................................................cgaauguugcucggugaac.............................   26      0    seq
    .....................................................cgaauguugcucggugaacc............................   24      0    seq
    .....................................................cgaauguugcucggugaaccc...........................   43      0    seq
    .....................................................cgaauguugcucggugaacccc..........................   266     0    seq
    .....................................................cgaauguugcucggugaaccccu.........................   1576    0    seq
    .....................................................cgaauguugcucggugaaccccuu........................   523     0    seq
    .....................................................cgaauguugcucggugaaccccuuu.......................   150     0    seq
    .....................................................cgaauguugcucggugaaccccuuuu......................   19      0    seq
    ......................................................gaauguugcucggugaac.............................   8       0    seq
    ......................................................gaauguugcucggugaacc............................   22      0    seq
    ......................................................gaauguugcucggugaaccc...........................   61      0    seq
    ......................................................gaauguugcucggugaacccc..........................   347     0    seq
```

oar-miR-409-5p

oar-miR-409-3p

ccucuccaugguacgcggggagagguuacccgagcaacuuugcaucuggacgacgaauguugcucggugaaccccuuuucgguaucaaaauccaucagaga

....gaauguugcucggugaaccccu.........................	4440	0	seq
....gaauguugcucggugaaccccuu........................	1684	0	seq
....gaauguugcucggugaaccccuuu.......................	1309	0	seq
....gaauguugcucggugaaccccuuuu......................	136	0	seq
....gaauguugcucggugaaccccuuuuc.....................	2	0	seq
....aauguugcucggugaacc............................	2	0	seq
....aauguugcucggugaaccc...........................	7	0	seq
....aauguugcucggugaacccc..........................	44	0	seq
....aauguugcucggugaaccccu.........................	844	0	seq
....aauguugcucggugaaccccuu........................	180	0	seq
....aauguugcucggugaaccccuuu.......................	138	0	seq
....aauguugcucggugaaccccuuuu......................	15	0	seq
....uguugcucggugaaccccuuu.......................	5	0	seq
....uguugcucggugaaccccuuuu......................	1	0	seq
